# Tracts on Generation

**Published:** 1847-12

**Authors:** 


### ARTICLE XII.

*Tracts on Generation. Number* 1. Proofs that the periodic maturation and discharge of ova, are in the mammalia and human female, independent of coition as a first condition of their propagation. By T. L. G. Bischoff, M. D., Professor of Physiology, &c., Giessen. Translated from the German by C. R. Gilman, M. D., Professor of Obstetrics, &c., College of Physicians and Surgeons, N. Y., and Theodore Tellkampf, M. D., Gebhard professor, Columbia College. pp. 65. S. S. & W. Wood, New York. (From the Publishers.)

This is an exceedingly interesting tract upon a subject that has, for the last few years, attracted a considerable amount of attention from physiologists.

A synopsis of the observations of the author was given in Vol. 2, first series, of this Journal, but as this is fuller and more in detail than the review from which it was taken, we shall extend the notice of its contents

Amongst the most important points that appear to be established by these researches, are, 1st. The actual contact of the semen masculinum with the ovum. 2d. That an ovum is discharged from the ovary, and transmitted through the fallopean tubes at each menstrual period, or the period of heat in the inferior animals.

In reference to the first of these propositions, it is sufficiently manifest from the microscopic observations of the author and others, that the spermatozoæ do reach the fallopean tubes, as they have been repeatedly found in that situation.

The following experiment and observations illustrate this point, as also the maturation of ova independent of coitus, in the interest of which will be found a sufficient reason for laying the facts a second time before our readers.

"With the intention of ascertaining to what point the male semen penetrates in bitches at the time of coition, I had kept a strong, healthy young bitch, which had never been covered. Since all depended upon my knowing, with absolute certainty, the time of the first coition, I kept her in my own house and watched her carefully. In the beginning of June, 1843, I observed that she was near the time of heat, the dogs began to follow her eagerly, and blood was discharged from the vagina; on Friday, June

9th, she did not allow herself to be covered. I then chained her up and isolated her strictly till the 11th, at three-quarters past one, when I put a dog to her, and she was covered for the first time. That this was a first coition was evident by her resistance and cries. As soon as coition was over, I extirpated the left uterus, ovary, and tube, and closed the wound by suture. I first examined the uterus, and found it quite full of spermatozoa in active motion. I intended next to examine the tube to find whether the semen had penetrated into it, but while preparing it, on laying bare the ovaries, I saw to my astonishment, that the ova which I had certainly expected to find in the Graafian Vesicle, had been already discharged from the ovary, for there were five small openings on the ovary, from one of which a little mass projected; five Graafian Vesicles had, therefore, already burst. The formation of the corpora lutea had even made some progress, commencing at the base and on the walls of the follicles; they even presented a considerable cavity filled with limpid serum, in which, however, no ovum was contained. It was now apparent to me how such a state of things had possibly led former observers, who were ignorant of the ovule, to the belief that the follicles were not yet opened. I gained at once the full conviction that they had opened, by finding the five ova near each other in the tube, about two inches from the fimbriæ. Nothing new resulted from their investigation; they had, in every respect, the characteristics which I had already seen in ova at this period of their development, and were entirely identical with the perfectly mature ovarian ovule. I looked in vain throughout the whole tube up to its uterine orifice, for spermatozoa; nowhere was a single one to be seen, and I spent so much time and care in this search, that I venture to assert most positively that the semen had not yet entered the tube. Next morning, at 10 o'clock, twenty hours later (within which time I had, in my former observations, usually found that the semen reached the ovary): I ordered this bitch to be killed. The right ovary showed five small openings, and five corpora lutea further developed, and in addition quite a large Graafian Vesicle, not yet ruptured; the tube contained five ova, which had progressed beyond its middle, and were several lines apart. Three of them were quite normal in their condition, and similar to those of yesterday, but two were plainly abnormal and abortive, the zona indistinct, the discus proligerus incompletely developed, the vitellus a small irregular mass of yolk granules. I now found spermatozoa in the tube, partly in motion, partly not; they had, however, penetrated not more than three lines beyond the uterine orifice. The whole remaining portion of the tube contained none, nor was there any vestige of them upon or around the ova; *the ova had evidently not been fecundated.*

"I believe that this observation incontestibly proves that the ova, when matured, leave the ovary and penetrate the tube, without any influence of the coitus. That it had not taken place before the time when I observed it, may be considered as certain, in view of the great care that had been taken; that the ova had been discharged by any influence of the coitus cannot for a moment be admitted, because,

"1st. It is certain that coition does not always produce this effect, as I myself have found that after coition had been frequently repeated, the Graafian Vesicles were still closed, and,

"2d. As it cannot be imagined that the ova had, in the short space of a quarter of an hour, passed over two inches of the narrow Fallopian tube, since it requires, as we know, eight days to pass the other two or three inches. If, therefore, quite independently of coition, the ova actually leave the ovary, and pass, unfecundated, into the tube, and may remain unfecundated after a period of twenty hours—we are first to inquire how this agrees with my former observations, in which I found in bitches, six, eighteen, or twenty hours after the first coition, the Graafian Vesicles still closed, and the semen then penetrated through the whole extent of the tube, and even upon the ovary? The answer of these inquiries evidently is, that when the ova are mature, fecundation is possible within certain limits of time and space. It depends, as it appears, on the peculiarities of the animal, and on the occurrence of opportunity, whether coition is consummated while the ova are still in the ovary or not till they are already detached and have entered the tube. Were animals placed in perfectly natural circumstances, and opportunity offered for coition, it appears probable that the sexual instinct would exhibit itself before the ova were discharged. If coition be at that time consummated, the semen may penetrate through the tubes to the ovary, and this, as my observations have shown, may take place in twenty hours. Other bitches admit the dog later, or, perhaps, opportunity is wanting, the animal being, as in the case above detailed, locked up,—then the ova are none the less detached, and may even after that, if coition take place, be fecundated; how long this is possible, I cannot say with certainty; but as bitches generally admit the male for the space of eight days, and as the first manifestations of development in the ovum, the division of the yolk, begins in the lower portion of the tube, where they are met with about the seventh or eighth day, it appears that this is the limit within which fecundation of the ovum is possible in the bitch."

The author makes numerous experiments upon rabbits, dogs, rats, &c., with a view of illustrating the formation of the corpora lutea, and the development of ova prior to the influence of fecundation upon them.

Several of these observations lead to the conclusion that fecundation generally takes place in the fallopian tubes, as the ova seem to go on in their development regularly until passed into them, and there perish unless met by the vivifying power of the semen masculinum. And, notwithstanding the spermatozoa are sometimes transmitted the whole length of the tubes, it does not necessarily follow that the fecundating influence is exerted prior to the discharge of the ovum from the Graafian Vesicle.

On this subject the author says:—

"After I had gained an insight into these phenomena, many of the results of my former experiments, which I had then considered as futile, or had differently interpreted, again engaged my attention. I had been, as I have mentioned, under the erroneous impression that coition was the cause of the discharge of the ovum from the ovary. I therefore counted always, as all my predecessors had done from the first coition; it accidently so happened that most of the bitches which were used for these observations were probably covered before the ova were discharged. Still, I find among my previous observations, several in which I had at the time noted that though the ova were found in the upper third of the tube, I saw spermatozoa only in its lower portion. As I, however, then knew that they did penetrate through the whole length of the tube, I supposed that I had overlooked them in the upper portions, perhaps because their number had been small, or that I had not been sufficiently careful in my experiments. I am now convinced that these were cases in which the ova had been discharged before coition had taken place, and the semen had time to penetrate further in the tubes. In bitches I had only seen spermatozoa *uniformly on the ova* in the lower third of the tube, rarely in the upper portions.

"In rabbits, where the period of heat cannot be as accurately ascertained as in bitches, and where the male generally improves the first opportunity for coition which the female will afford, it appears that the ova are not usually discharged till the semen has time to reach the ovary, this requiring, according to the observations of Barry and myself, nine or ten hours. In rabbits I have always found spermatozoa on the ova in the upper third of the tube; and with them the division of the yolk begins higher up in the tube, and the limits within which fecundation is possible are probably much shorter than in the bitch."

